# Pathogenesis and therapeutic applications of microglia receptors in Alzheimer’s disease

**DOI:** 10.3389/fimmu.2025.1508023

**Published:** 2025-02-14

**Authors:** Jiao Fu, RuoXuan Wang, JiHui He, XiaoJing Liu, XinXin Wang, JuMing Yao, Ye Liu, ChongZhao Ran, QingSong Ye, Yan He

**Affiliations:** ^1^ Institute of Regenerative and Translational Medicine, Tianyou Hospital, Wuhan University of Science and Technology, Wuhan, China; ^2^ First Clinical College, Wuhan University of Science and Technology, Wuhan, China; ^3^ Center of Regenerative Medicine, Renmin Hospital of Wuhan University, Wuhan, China; ^4^ Athinoula A. Martinos Center for Biomedical Imaging, Massachusetts General Hospital and Harvard Medical School, Boston, MA, United States; ^5^ Department of Stomatology, Tianyou Hospital, Wuhan University of Science and Technology, Wuhan, China

**Keywords:** microglia receptor, microglia, Alzheimer’s disease, neuroinflammation, heterogeneity

## Abstract

Microglia, the resident immune cells of the central nervous system, continuously monitor the brain’s microenvironment through their array of specific receptors. Once brain function is altered, microglia are recruited to specific sites to perform their immune functions, including phagocytosis of misfolded proteins, cellular debris, and apoptotic cells to maintain homeostasis. When toxic substances are overproduced, microglia are over-activated to produce large amounts of pro-inflammatory cytokines, which induce chronic inflammatory responses and lead to neurotoxicity. Additionally, microglia can also monitor and protect neuronal function through microglia-neuron crosstalk. Microglia receptors are important mediators for microglia to receive external stimuli, regulate the functional state of microglia, and transmit signals between cells. In this paper, we first review the role of microglia-expressed receptors in the pathogenesis and treatment of Alzheimer’s disease; moreover, we emphasize the complexity of targeting microglia for therapeutic interventions in neurodegenerative disorders to inform the discovery of new biomarkers and the development of innovative therapeutics

## 1 Introduction

Alzheimer’s disease (AD) is the most common neurodegenerative cause of dementia, accounting for 60-80% of dementia cases. According to the World Alzheimer’s Report 2024, approximately 6.9 million older Americans aged 65 and older currently have the disease. This number will increase to 13.8 million by 2060 if significant progress is not made in the prevention, mitigation, or treatment of AD (1). The main neuropathological hallmarks of AD include the extracellular aggregation of amyloid-beta (Aβ) peptides into plaques and the intracellular accumulation of hyperphosphorylated tau protein, resulting in neurofibrillary tangles (NFTs) (2). Indeed, AD pathogenesis is now recognized as a complex array of pathogenic processes, a variety of factors including genetics, amyloid, tau, ApoE, neuroimmune activation, and infection (3).These factors can lead to synaptic dysfunction and neuronal loss, ultimately causing cognitive impairment (4).

With the rapid development of immunotherapy and the exploration of new therapeutic targets for AD in recent years, microglia have received widespread attention for their role in treating AD. These innate immune cells, equipped with receptors that sense brain changes, have been confirmed to be associated with AD risk by genome-wide association studies (GWAS) (5). Under physiological conditions, microglia maintain brain homeostasis by phagocytosing neuronal synapses, apoptotic cells, and cellular debris, and by releasing trophic factors that support neuronal development and survival (6). However, overactivation of microglia under conditions of stimulation or disease leads to morphological changes and abnormal production of cytokines, which may exacerbate neuroinflammation with consequent effects on the progression of AD (7). Targeting the aberrant activation of microglia presents a novel strategy for curbing neuroinflammation and treating central nervous system (CNS) disorders (8). The phenotypic regulation of microglia is closely linked to pattern recognition receptors (PRRs) that recognize Aβ, pathogen-associated molecular patterns (PAMPs), and other damage-associated molecular patterns (DAMPs), which in turn affect microglial function and phenotype (9). While the function of microglial receptors has been studied, the comprehensive mechanisms of their regulation of microglial function remain unclear. In this paper, we will analyze the receptor-ligand interaction signaling axis that governs microglial function and evaluate its role in AD pathogenesis and potential therapeutics (**Table 1**).

**Table 1:** Microglia Receptors involving aggregation clearance and immune modulation.

Receptor family	Receptor	Stimulate	Functions	Treatment Strategies	References
IgSF	TREM2	Aβ tau ApoE LPS	1) Mediates Aβ phagocytosis 2) Modulates immune response to reduce inflammation 3) Regulates autophagy to promote cell survival	Anti-TREM2 antibodies: AL-OO2 and DNL919	(10, 11)
Siglecs	CD33	Aβ	1) Involved in Aβ clearance 2) Modulates microglial response to mitigate inflammation	anti-CD33 antibody: HuM195	(12)
RTK	TAM	Aβ tau	1) Contributes to plaque modification and removal	Aβ phagocytosis inducer: αAβ-Gas6	(13, 14)
SR	SR-A	Aβ oxLDL pathogens	1) Involved in Aβ clearance 2) Attenuates inflammatory responses	/	(15)
SR	CD36	Aβ oxLDL pathogens	1) Involved in Aβ clearance 2) Upregulation of IL-1β and NO production through the NF-κB and MAPK pathways	CD36 inhibitors: salvinorin B, tanshinone IIA, curcumin, and small molecule compounds	(16, 17)
LILRB	LilrB	APOE4	1) LilrB3 activates microglia to enter a pro-inflammatory state 2) LilrB4 inhibits phagocytosis	Anti-LilrB4 antibody	(18, 19)
NLR	NLRP3	Aβ tau pathogens	1) Promote the maturation of the inflammatory cytokines IL-1β and IL-18	NLRP3 inhibitors: MCC950 and UK5099	(20)
PRRs	RAGE	Aβ tau LPS	1) Implicated in the abnormal aggregation of pathological proteins 2) Activation of the NLRP3 inflammasome promotes the secretion of IL-1β and the release of the N-GSDMD	RAGE inhibitors: Azeliragon and FPS-ZM1	(21)
PRRs	Dectin-1	Aβ	1) Triggers the Syk/NF-κB signaling cascade and stimulates the expression of inflammatory factors	Anti-CLEC7A antibody	(22)
PRRs	TLRs	Aβ tau LPS	1) Recognizes PAMPs and DAMPs, triggers NF-κB and additional transcription factors, and enhances the generation of pro-inflammatory cytokines.	TLR antagonists: TAK-242, GX-50 and baicalein.	(23)
NRs	PPAR	Aβ tau LPS	1) PPAR-α activation reprograms the immune response 2) PPAR-γ activation reduces inflammation	PPAR agonist: DTMB and T3D-959	(24, 25)
GPCR	GluRs	Glutamate	1) mGluR2 activation promotes neurotoxicity 2) mGluR5 activation shows neuroprotection	mGluR2 inhibitor MCCG mGluR5 agonist CHPG	(26, 27)
LGICs	P2XR	Aβ tau ApoE ATP	1) Influence the clearance of Aβ and tau 2) Promoting inflammatory responses by regulating the expression of NLRP3 inflammasomes and chemokines	P2×7R antagonists: Brilliant blue G and AZ10606120	(28, 29)
Chemokine receptor	CX3CR1	Aβ tau CX3CL1	1) Influence the clearance of Aβ and tau 2) Regulate the distribution, migration and function of microglia	/	(30, 31)
PDGFR	CSF1R	Aβ tau	1) Promoting Aβ plaque formation 2) Influence the clearance of tau	CSF1R inhibitors: GW2580 and PLX5622	(32, 33)
unknown	AdipoR1	adiponectin	1) Modulates microglial response to limit inflammation	adiponectin receptor agonist: AdipoRon	(34)

IgSF, immunoglobulin superfamily; Siglecs, sialic acid-binding immunoglobulin-like lectins; RTK, receptor tyrosine kinase; SR, scavenger receptor; LILRB, leukocyte immunoglobulin-like receptors B; NLR, NOD-like receptor; PRRs, pattern recognition receptors; NR, nuclear receptor superfamily; GPCR, G protein-coupled receptors; LGICs, ligand-gated ion channels; PDGFR, platelet-derived growth factor receptor.

## 2 Physiology and pathology of microglia in AD

Microglia originate from the early yolk sac and are the primary immune cells of the CNS. These cells enter the CNS during early embryonic development and become an intrinsic immune cell that provides immune surveillance of the CNS (35). In their resting state, microglia exhibit a branched morphology. These elongated branching structures enable them to perform extensive surveillance in CNS tissues, a key feature that distinguishes them from macrophages.

Microglia possess a variety of biological properties, such as pathogen phagocytosis, cellular debris removal, and inflammatory factor secretion, which play important roles in the CNS (36). However, the functional state of microglia does not exist in isolation but is highly dependent on the microenvironment in which they reside (37). Studies have shown that even in the healthy brain, microglia populations display complex heterogeneity and are subject to a combination of factors such as gender, age, circadian rhythms, gut flora, the central nervous system, the surrounding environment, and disease state (38, 39). Nevertheless, microglia are always actively monitoring the CNS microenvironment and swiftly adapt their phenotype upon detecting injury signals (40, 41). Microglia sensory bodies composed of various receptors and signaling molecules enable them to sensitively detect environmental changes and respond adaptively to maintain CNS homeostasis (42, 43). This dynamic interaction with the CNS microenvironment is especially critical in neurodegenerative diseases like AD.

In AD, microglia recognize pathogens, cellular debris, or abnormal proteins (including misfolded Aβ and tau) through their surface receptors and induce microglial responses (44). Activated microglia internalize pathogenic substances through pinocytosis, phagocytosis, or receptor-mediated endocytosis and attempt to degrade them via various endocytic pathways, such as the lysosomal pathway, as well as by activating the expression of relevant gene modules (45). This initial response is generally neuroprotective, aiming to eliminate harmful substances and maintain CNS integrity. However, as age and AD pathology progress, microglia’s ability to address brain challenges, such as toxic amyloid, infection, and compromised neurons, becomes compromised (46, 47). Microglia often become dysfunctional and susceptible to sustained activation, leading to neurotoxicity and synapse loss (48, 49). Sustained microglial activation also impairs their ability to effectively clear Aβ and tau tangles, alongside the generation of numerous toxic substances, particularly pro-inflammatory cytokines and chemokines (50). This dysregulated microglial response contributes to the neuroinflammation and neurodegeneration characteristic of AD. Therefore, understanding the role of microglia and their receptors in AD is essential for developing therapeutic strategies to modulate microglial activity and attenuate the disease process.

## 3 Receptors involving aggregation clearance and microenvironment regulation of microglia

**Figure 1:**
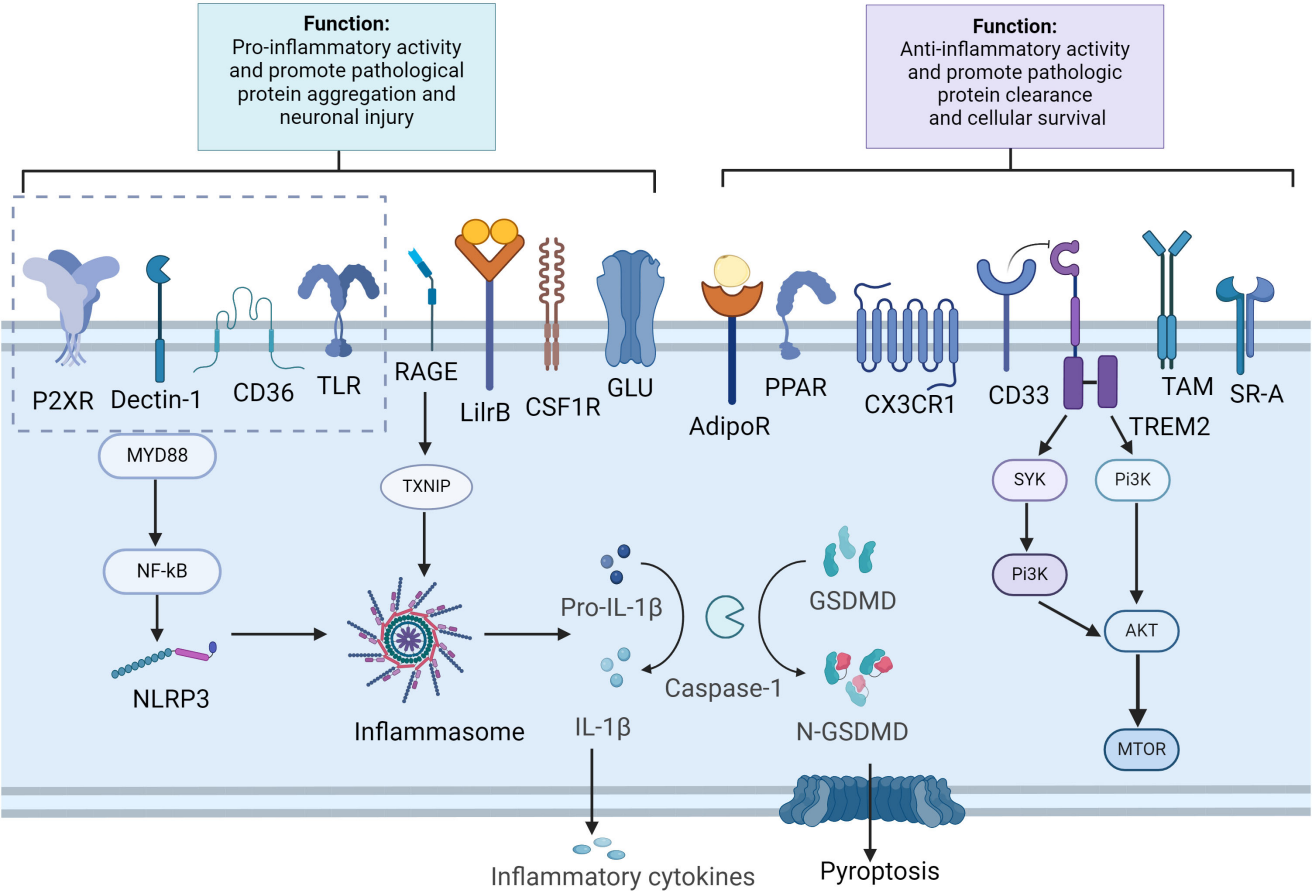
Microglia receptors and signal pathways in aggregation clearance and immune modulation. Created in BioRender.com. In Alzheimer’s disease, pathological substances such as Aβ and tau can activate microglial cells through various receptors, including CD36, TLRs, RAGE, P2XR and Dectin-1. This activation leads to the stimulation of the NF-κB, which in turn triggers the activation of the inflammasome complex. This results in the cleavage of pro-caspase-1 into active caspase-1 and the subsequent processing of pro-IL1β and GSDMD. The release of inflammatory cytokines and the cleavage of GSDMD into N-GSDMD lead to the formation of pores in the plasma membrane, causing pyroptosis in microglia. Furthermore, the activation of receptors such as AdipoR1, TAM receptors, CX3CR1, CD33, TREM2, PPAR, and SR-A aids in the clearance of pathological substances and mitigates neuroinflammation. Additionally, TREM2 activation, proliferation and phagocytosis by activating the mTOR pathway. AdipoR, adiponectin receptors; GSDMD, Gasdermin D; LilrB, leukocyte immunoglobulin-like receptors B; mTOR, mammalian target of rapamycin; N-GSDMD, N-terminal GSDMD; PPAR, peroxisome proliferator-activated receptor; NLRP3, NLR family pyrin domain containing 3; RAGE, receptor for advanced glycation end products; SR-A, scavenger receptor A; TAM, tyrosine-based activation motif; TLR, toll-like receptor; TXNIP, thioredoxin-interacting protein; TREM2, triggering receptor expressed on myeloid cells 2.

Structural and functional changes in microglial receptors play a key role in the pathologic process of AD (**Figure 1**). Microglia are involved in the phagocytosis of aberrant proteins, such as misfolded Aβ and tau, through the expression of multiple receptor families. These receptors include immunomodulatory receptors like TREM2 and CD33 (51, 52), scavenger receptors such as SR-A and CD36 (53, 54), and receptor tyrosine kinases of the TAM family—Tyro3, Axl, and MerTK (55). Microglia also express a diverse set of pattern recognition receptors (PRRs), including Toll-like receptors (TLRs), NOD-like receptors (NLRs), and C-type lectin receptors (CLRs), which enable the recognition of endogenous neurotoxic ligands, notably Aβ and tau (56–59). Additionally, they possess neurotransmitter receptors, such as those for glutamate and purines, that facilitate interactions between neurons and microglia (60, 61).

Once microglia were thought to be homogeneous cells that responded consistently to their environment. However, recent developments in single-cell technology have revealed a variety of microglia states in normal and diseased brains (62). Microglial phenotypes are intricately regulated by a complex network of signaling pathways and transcription factors, which modulate their functional states in response to environmental cues (63). For example, TREM2 signaling is crucial for maintaining microglial homeostasis and supporting phagocytic functions (64). However, under AD pathological conditions, functional variants of TREM2 (e.g., the R47H mutation) impair their ability to phagocytose Aβ, thereby promoting disease progression (65). This highlights the importance of understanding the broader regulatory network in which microglial receptors operate. Thus, a deeper understanding of the mechanisms of microglial receptors in AD and how to modulate microglial function by targeting these receptors is critical for effective intervention in the disease process. Below, we have selected some of the more hotly studied and novel microglial receptors and described their functions and differential effects in AD.

### 3.1 Triggering receptor expressed on myeloid cells 2

TREM2 is a transmembrane receptor with immunomodulatory functions characterized by a V-type Ig structural domain, transmembrane helices, and short cytoplasmic tails.TREM2 has been shown to interact with various ligands, including Aβ, apoptotic cells, and lipid-related ligands such as ApoE (66, 67). These interactions are critical for maintaining nervous system homeostasis. Specifically, upon ligand binding, TREM2 induces the phosphorylation of DAP12, which subsequently recruits and activates spleen tyrosine kinase (SYK). This signaling cascade to regulate microglial phagocytosis, proliferation, survival, immune responses, energy metabolism, and autophagy (68).

Polymorphisms in the TREM2 gene are major risk factors for neurodegenerative disorders like Alzheimer’s disease (69, 70). In particular, the R47H mutation in the coding region of TREM2 increased the susceptibility to AD, with an increased risk of up to 4.5-fold (71), thus establishing TREM2 as an immunogenetic risk factor in AD. Recent studies have explored the signaling properties of the TREM2R47H variant through a mouse model (72, 73). Under normal conditions, microglial binding to Aβ induces a transcriptional signature known as disease-associated microglia (DAM), primarily mediated by the TREM2-DAP12 receptor complex. This complex transmits critical intracellular signals via SYK, modulating microglial phagocytosis and inflammatory responses (74). The TREM2R47H variant impairs the ability of microglia to encapsulate Aβ plaques and promotes Aβ plaque spreading and neurotoxicity as well as AD progression (75).

Additionally, TREM2 is involved in lipid metabolism (76). It senses lipids and mediates myelin phagocytosis. TREM2 dysfunction results in the accumulation of pathologic lipids in microglia, likely because TREM2 deficiency impairs the ability of microglial cells to transition to DAM, which effectively removes myelin cholesterol. Enhancing TREM2 function reduces cholesterol and cholesteryl ester (CE) load in microglia, in part by facilitating lipid clearance (77). TREM2 also regulates microglial autophagy and promotes microglial cell survival by activating the PI3K/AKT/mTOR pathway (78). Overall, TREM2 enhances microglial phagocytosis in response to inflammatory stimuli, thereby attenuating AD-related pathologic changes. However, the role of TREM2 is not limited to neurodegenerative diseases and may be dual in different disease contexts. For example, in certain types of tumors, TREM2 may promote tumor immune escape. In gliomas, knockdown of TREM2 expression in microglia may inhibit tumor cell proliferation and contribute to delaying tumor progression (79). These findings suggest that TREM2 may play an inhibitory role in the tumor microenvironment, suppressing the immune surveillance function of microglia. Thus, the functions of TREM2 may differ in different disease contexts, and further in-depth studies are needed to investigate the complex mechanisms of TREM2’s role in AD and other disorders.

### 3.2 Cluster of differentiation 33

CD33 is an inhibitory immune receptor that belongs to the family of Sialic Acid-Binding Immunoglobulin-Like Lectins (Siglecs) (80). It possesses an immunoreceptor tyrosine-based inhibitory motif (ITIM) that, upon phosphorylation, attracts protein tyrosine phosphatases such as SHP-1 and SHP-2, thereby suppressing cell activation, proliferation, cytokine production, and phagocytosis (81, 82). In 2011, two genome-wide association studies (GWAS) found that the single nucleotide polymorphism (SNP) rs3865444, located upstream of CD33, was associated with AD risk (83). In the central nervous system, CD33 is specifically expressed on microglial cells. Its potential link to AD susceptibility has drawn significant attention from the scientific community (84). Studies have shown CD33 levels are negatively correlated with Aβ clearance. The variant of CD33 associated with an increased risk of AD is expressed at relatively higher levels, which inhibits the phagocytic function of CD33 towards Aβ (85). In a CD33 knockout (CD33-/-) mouse model, the lack of CD33 was linked to decreased Aβ levels and a reduction in amyloid plaques in the brain (86). Moreover, Wong et al. demonstrated that targeting CD33 with HuM195, an anti-CD33 antibody, and its single-chain variable fragments can enhance Aβ phagocytosis by microglia by blocking CD33’s inhibitory signaling (87). Consequently, this finding implies that diminished levels of CD33 expression could potentially empower microglia to more effectively eliminate detrimental Aβ peptides. To investigate the role of human CD33 isoforms in microglial function, given the functional differences between mice and humans, researchers have developed transgenic mouse models expressing different hCD33 isoforms. Eskandari et al. demonstrated that, compared to 5XFAD control mice, mice expressing the long form of the CD33 isoform (CD33M) exhibited higher levels of Aβ, more diffuse plaques, reduced disease-associated microglia, and more dystrophic neuromasts (12). In contrast, mice that express the short CD33 isoform (CD33m) exhibited plaque compression, enhanced microglia-plaque contacts, and minimized neuroinflammatory plaque pathology, highlighting the protective role of the CD33m isoform in AD (12). Of particular importance, the protective phenotype driven by CD33m isoforms was observed at an early stage of the disease, suggesting that CD33m has a significant regulatory role on microglia function early in disease progression. These findings emphasize the central role of CD33 in the neuropathological mechanisms of AD and place it as a notable hotspot in AD research, providing a promising target for possible future therapeutic interventions.

### 3.3 Tyrosine-activated motif receptors

The TAM receptor family, which includes tyrosine kinase receptors such as Tyro3, Axl, and MerTK, plays a key role in regulating phagocytosis and inflammatory responses in microglia (88). Under physiological conditions, human and mouse microglia highly express MerTK and low levels of Axl, but not Tyro3 (89). In AD patients and mouse models, Axl and MerTK expression is elevated in microglia associated with amyloid plaques, where they contribute to plaque modification and clearance by binding to Gas6 and phosphatidylserine (PtdSer) (13). This process is crucial for maintaining brain environment stability and slowing AD progression. In the APP/PS1 mouse model, the absence of Axl and MerTK genes impaired microglial recognition and phagocytosis of amyloid β plaques, highlighting the TAM system’s essential role in these processes. TAM receptor activation also compresses Aβ plaques, potentially as a protective mechanism to minimize brain damage (14).

Furthermore, a separate study reveals a microglial phagocytic deficit. Specifically, tau can induce the expression of MerTK in microglia, which then recognize and engulf neurons exposing PtdSer through Gas6, thereby promoting neuronal loss (90). Notably, αAβ-Gas6, a novel fusion protein, selectively clears Aβ in a TAM receptor-dependent manner without inducing NF-kB-mediated inflammation (91). This protein effectively scavenges Aβ and avoids the inflammatory response triggered by conventional antibodies, offering novel therapeutic strategies for AD treatment.

### 3.4 Scavenger receptor A

SR-A is a pattern recognition receptor expressed mainly in myeloid cells and plays an important role in the natural immune system. It is expressed in CNS, microglia, and astrocytes (92). SR-A has a variety of biological roles, such as the clearance of pathogens, apoptotic cells, and lipids (93). SR-A deficiency leads to a decrease in microglial inflammatory response and phagocytosis (94). As animals age, the SR-A expressed in microglia decreases, which may affect their scavenging and phagocytosis of Aβ. Cornejo et al. found that SR-A expression was reduced in the brains of aging animals, and SR-A knockout resulted in the release of more nitric oxide (NO) and proinflammatory cytokines from mouse microglia, reduced production of anti-inflammatory cytokines, and decreased Aβ phagocytic activity, which leads to further deposition of Aβ in the aging brain and promotes the development of AD (15). In an SR-A-deficient mouse model of AD, microglia-mediated phagocytosis of soluble Aβ was reduced, and the expression levels of enkephalinase and insulin-like growth factor 1 (IGF-1), which have been reported to be Aβ-degrading enzymes in AD, were also significantly reduced, suggesting that SR-A may alleviate AD by affecting Aβ phagocytosis (95). Conversely, overexpression of SR-A reduces Aβ levels and promotes Aβ clearance (96). However, despite the potential that SR-A shows in AD therapy, no drugs targeting SR-A in microglia have been discovered and developed.

### 3.5 Cluster of differentiation 36

As an SR-B receptor, CD36 is one of the key receptors mediating the phagocytic response of microglia. In animal models of AD, amyloid binds to CD36 and attempts to eliminate Aβ deposits by inducing CD36 expression and stimulating phagocytosis (97). However, this process seems to trigger an inflammatory response, which specifically seems to say that the binding of CD36 to Aβ activates the inflammasome NOD-like receptor family pyrin structural domain-containing 3 (NLRP3), which promotes the release of proinflammatory cytokines, chemokines, and ROS, exacerbates neuroinflammation, and leads to a progressive worsening of AD (16). In this case, CD36 seems to do more harm than good. In addition, CD36 expression is regulated by a variety of proteins, including PPAR, NgR, and others. A study has shown that CD36 can be upregulated by selective PPAR agonists, leading to increased microglia accumulation and microglial Aβ phagocytosis in and around Aβ plaques, as demonstrated in the APP/PS1 transgenic mouse model (17). In contrast, the Nogo/NgR signaling pathway was shown to significantly reduce CD36 expression in adult microglia (98). The association between the Nogo receptor and CD36 expression was further confirmed by Wang et al. (99), who proposed that enhancing the expression of the Nogo receptor could inhibit the transcription of the CD36 gene, thereby reducing the phagocytosis of Aβ by microglia. Although it seems contradictory on the role of CD36 in AD pathology, in general, AD therapeutic strategies targeting CD36 have focused on two aspects: 1) blocking CD36 using neutralizing antibodies or other small molecules to inhibit the inflammatory response of microglia, such as salvinorin B, tanshinone IIA, curcumin, and small molecule compounds (100). and 2) enhancing Aβ clearance by upregulating CD36 expression. However, the exact mechanism and balance of these two approaches still need further studies to elucidate.

### 3.6 Leukocyte immunoglobulin-like receptors B

LilrB3 is a specific cell surface receptor for APOE4, as revealed in a study by Shi et al. (18). APOE4 is the strongest known genetic risk factor for LOAD, and LilrB3 acts as an immune checkpoint receptor protein that binds specifically to APOE4 and almost not to APOE2. This specific binding is capable of activating microglia, prompting a shift to a pro-inflammatory state, and providing new molecular-level insights into the pathogenesis of AD (101). The team further resolved the high-resolution structure of the LilrB3-APOE4 complex by cryo-electron microscopy. It was found that the extracellular structural domain (ECD) of LilrB3 contains two discontinuous immunoglobulin-like structural domains that recognize and bind to positively charged surface patch on the N-terminal structural domain (NTD) of APOE4. The revelation of this structural detail provides evidence at the molecular level for understanding how APOE4 activates microglia through the LilrB3 receptor. This binding brings the intracellular signaling motifs of the LilrB3 molecules in close proximity to each other, triggering microglia (e.g., the HMC3 cell line) to enter a pro-inflammatory state (18). This process is consistent with the LilrB3-dependent activation of APOE4 but not APOE2, revealing a unique role for APOE4 in immune regulation. This finding is scientifically important for unraveling the functional differences between different isoforms of the APOE gene and how they affect the onset and progression of related diseases, especially Alzheimer’s disease.

Furthermore, LilrB4 also plays a role in regulating microglia function in AD. LilrB4 interacts with membrane-bound ligands and signals through an immunoreceptor tyrosine-based inhibitory motif (ITIM) in its cytoplasmic domain (19). In AD, high LilrB4 expression in activated microglia inhibits phagocytosis (102, 103). Recently, Hou et al. investigated antagonist antibodies targeting human leukocyte Ig-like receptor B4 to enhance microglia responses. In an AD mouse model, human LilrB4 expression reduced the association between microglia and amyloid plaques, increasing amyloid pathology. This effect was reversed by LilrB4-specific antibody treatment, which enhanced microglia phagocytosis of amyloid plaques, suggesting that LilrB4 is a potential therapeutic target for AD (104).

### 3.7 NLR family pyrin domain containing 3

In the neuropathology of AD, the NLRP3 inflammatory vesicle serves as a key pattern recognition receptor in the CNS and is expressed predominantly in microglia (105). The NLRP3 inflammasome is strongly activated in AD and may contribute to the pathogenesis of the disease. Activation of the NLRP3 inflammasome is a complex molecular event involving a sensor (NLRP3), a junction protein (e.g., ASC or PYCARD), and an effector molecule (e.g., caspase-1) (106). Specifically, activation of the NLRP3 inflammasome leads to the conversion of the precursor caspase-1 to its active form, and then, by its protease activity, activation of caspase-1 drives the cleavage, maturation, and release of cytokine precursors such as IL-1β and IL-18, which ultimately leads to the inflammatory necrosis of cells (e.g., neurons, glial cells, etc.) (20). In addition to causing pro-inflammatory factor activation and release, NLRP3 inflammatory vesicle activity leads to the release of assembled ASC speckles. Some studies have shown that ASC specks released by microglia bind rapidly to amyloid-β and increase the formation of Aβ oligomers and aggregates, acting as an inflammation-driven cross-seed for Aβ pathology (107, 108). This suggests that ASC plays a key role in the seeding and spread of Aβ pathology. Moreover, genetic studies have shown that caspase-1 shearing, reduced IL-1β activation, enhanced Aβ clearance, and enhanced learning memory were found in APP/PS1/NLRP3-/- mouse models (109, 110). Notably, NLRP3 inflammatory vesicles can be activated both by Aβ aggregation and by small-molecule Aβ oligomers and protofibrils, suggesting that the central intrinsic immune response is initiated before Aβ deposition (111). In addition, gut flora transplantation in AD patients upregulates intestinal NLRP3 expression and peripheral blood levels of IL-18 and IL-1β in APP/PS1 double-transgenic mice, suggesting that NLRP3 signaling in peripheral inflammation may be transferred to the CNS, triggering neuroinflammation and other AD pathologies (112).

Moreover, tau proteins and their oligomers are strongly DAMPs-active and can be activated through the ASC-mediated activation of NLRP3-ASC inflammatory vesicles and promote IL-1β release (113). Inhibition of NLRP3 inflammatory vesicle expression in microglia can normalize Aβ metabolic pathways and reduce neuronal tau protein phosphorylation (114). In therapeutic strategies for AD, NLRP3 inflammasome inhibitors such as MCC950 show the potential to block NLRP3 inflammasome activation and reverse tau pathology (115). In addition, natural compounds such as acacetin inhibit NLRP3 inflammasome activation by decreasing ROS production and inhibiting ASC aggregation, showing potential as therapeutic candidates for AD (116).

### 3.8 Receptor for advanced glycation end-products

RAGE is expressed on a variety of immune and non-immune cells, including microglia, blood-brain barrier endothelial cells, and neurons (117). RAGE is closely associated with AD pathology. Notably, RAGE mediates the intraneuronal transport of Aβ, contributing to its accumulation and dissemination within neurons (118). This highlights that the effect of RAGE blockage on pathology propagation is likely mediated not only by immune cells but also by neurons themselves.

Overexpression of RAGE on microglia increases inflammatory responses and Aβ aggregation in transgenic mouse models of amyloid pathology (21). Recent studies have further elucidated the mechanism of RAGE in Aβ-mediated microglial activation. The RAGE-TXNIP axis formed by RAGE and Thioredoxin Interacting Protein (TXNIP) induces Aβ translocation from the cell surface to mitochondria, a process that is essential for the activation of NLRP3 inflammatory vesicles in mitochondria. It is known that NLRP3 inflammatory vesicles in mitochondria can promote IL-1β secretion and activate Gasdermin D (GSDMD), a protein associated with cellular death (119). By silencing TXNIP or inhibiting the activation of RAGE, the translocation of Aβ to mitochondria can be reduced, mitochondrial function can be restored, and the toxic effects of Aβ can be attenuated (120). Furthermore, RAGE also serves as a receptor for tau protein and is involved in the uptake and regulation of tau protein. Kim et al. found that the knockout of RAGE reduced the uptake of tau by microglia and neurons and slowed the propagation of tau between neurons. Treatment with the RAGE antagonist FPS-ZM1 blocked trans-synaptic tau propagation and inflammatory responses in rTg4510 mice and alleviated cognitive impairments (121). These suggest that RAGE not only plays a role in Aβ-associated neuroinflammation but may also be involved in tau protein-associated pathological processes, providing a new perspective on the complex pathological mechanisms of AD.

### 3.9 Dectin-1 receptor

The Dectin-1 receptor, encoded by the Clec7a gene, is a C-type lectin pattern-recognition receptor (CLR), and Clec7a expression is significantly upregulated in microglia associated with Aβ plaque deposition in mouse models and human AD brain tissues (122, 123). Dectin-1’s extracellular C-type lectin structural domain endows it with the ability to recognize and bind specific ligands (124). It can mediate the pro-inflammatory response to Aβ. Direct interaction of Aβ42 with Dectin-1 triggers homodimerization of the receptor and further activates downstream signaling pathways, such as splenic tyrosine kinase (Syk) and nuclear factor-κB (NF-κB), which promotes the secretion of pro-inflammatory cytokines and the production of reactive oxygen species (ROS) (22).

Dectin-1 is also crucial for microglial phagocytosis and is linked to TREM2. Specifically, TREM2 increases the expression of Dectin-1/Clec7a, as validated by RNA sequencing analysis (122). Clec7a activation can correct microglial phagocytosis deficiencies related to TREM2 mutations. Both TREM2 and Clec7a stimulate the downstream SYK signaling pathway, which promotes microglial activation as disease-associated microglia for Aβ plaque clearance (125). Colonna et al. discovered that in TREM2R47H mutant mice, which cannot activate SYK, treatment with a Clec7a-activating antibody can activate SYK, thus enhancing microglia’s capacity to clear Aβ plaques (75). This research indicates that Clec7a activation can partially compensate for the lack of SYK activation due to TREM2 mutations.

### 3.10 Toll-like receptors

In the human brain, microglia express a wide range of Toll-like receptors (TLRs), including TLR1 through TLR9 (126), of which TLR2 and TLR4 are considered to be the major functional subtypes in AD (127, 128). It has been shown that the receptor complex of microglia recognizing Aβ protofibrils contains TLR2, TLR4, and the co-receptor CD14 and that these components are essential for receptor function (129, 130). In terms of signaling, members of the TLRs typically utilize MyD88-dependent pathways for signaling, except TLR3 through the TRIF pathway. Activation of TLR2 and TLR4 triggers the activation of key transcription factors, such as NF-κB and AP1, through the MyD88 pathway, which further regulates the expression of inflammatory factors, chemokines, and co-stimulatory factors (131). TLR4 is an important receptor for inflammatory responses in the CNS and is closely related to the development of AD (132). Ligands such as Aβ and LPS can upregulate several inflammation-related genes by activating the TLR4-MyD88 signaling pathway, which in turn activates its downstream NF-κB, MAPKs, etc. (133, 134). Therapeutically, small-molecule drugs targeting TLR4 inhibitors such as TAK-242, GX-50, baicalein, etc. can significantly reduce neuroinflammation and neuronal damage by interfering with the TLR4 signaling pathway, thereby improving cognitive function in AD mouse models (23). In addition, tau protofibrils induced TLR2 activation in microglia, and tau protofibrils stimulated microglial inflammation via TLR2. It has been shown that rTg4510 tau transgenic mice knocked down for TLR2 reduced tau pathology and microglial activation (135). Similarly, in the PS19 mouse model, genetic deletion of TLR2 prevented the development of tau pathology and was accompanied by significant suppression of neuroinflammation as well as improved cognitive behavior (136). Although these data suggest that knockout of TLRs may provide novel strategies for AD treatment, MyD88-deficient mouse models show reduced Aβ load in the brain and amelioration of behavioral deficits (137). Furthermore, transplantation of MyD88-deficient myeloid cells is more effective at ameliorating brain Aβ levels and cognitive deficits compared with MyD88-normal myeloid cells in an AD mouse model (138). These findings suggest that the activation of TLRs may adversely affect the progression of AD, and therefore, the study of the TLR signaling pathway is important for the pathogenesis and prevention of AD.

### 3.11 Peroxisome proliferator-activated receptor

PPAR is a nuclear receptor, which is divided into three subtypes: PPAR-α, PPAR-γ, and PPAR-δ. Among them, PPAR-α and PPAR-γ have received more attention. They are significantly expressed in microglia and play a key role in the pathogenesis of AD. The activation of PPAR-α is closely associated with the recruitment of microglia and their phagocytosis of Aβ. In an AD transgenic mouse model, the PPAR-α agonist Gemfibrozil has been shown to enhance microglia autophagy, contributing to the removal of damaged organelles and accumulated proteins (139). In addition, the novel PPAR agonist DTMB inhibited neuroinflammation by decreasing the levels of NF-κB protein and reducing the production of pro-inflammatory cytokines by microglia. In the 5xFAD mouse model, DTMB not only improved learning and memory abilities but also reduced the formation of Aβ plaques in the brain. This reduction may be associated with reduced levels of neuroglial proliferation and chronic inflammation (24). Given the ability of PPAR-α agonists to cross the blood-brain barrier, they have the potential to be a novel strategy for the treatment of AD. On the other hand, PPAR-γ, a member of the nuclear receptor superfamily, is also significantly expressed in microglia and has important implications for the pathogenesis of AD (140). PPAR-γ agonists reduce the release of proinflammatory factors by inhibiting the activation of microglia and astrocytes (141). For example, the PPAR-γ activation was able to reduce neuroinflammation in AD by inhibiting microglia hyperactivation through the NF-κB signaling pathway (142). It was also found that PPAR-γ agonists were able to ameliorate learning and memory deficits in a mouse model of dementia by increasing the expression of low-density lipoprotein receptor-related protein 1 (LRP1) in the hippocampus (143). LRP1 is an Aβ-scavenging receptor, and its high expression reduces Aβ accumulation in the brain (144). Recent studies have further revealed the role of PPAR-γ in regulating microglia autophagy. The activation of PPAR-γ enhances mitochondrial autophagy and ameliorates cognitive deficits in APP/PS1 mice (25). Since autophagy dysfunction causes the accumulation of Aβ and tau proteins, PPAR-γ regulation of autophagy makes it a new target for AD prevention and treatment.

### 3.12 Glutamate receptors

GluRs are the most important receptors for the excitatory neurotransmitters within the central nervous system, and the overactivation of the GluR signaling can lead to excitotoxicity, which poses a threat to neuronal cell survival (145). Ionic and metabotropic glutamate receptors (GLU receptors) expressed by microglia play a complex role in the pathology of AD (146). The former receptors are classified into AMPA receptors, kainate (KA) receptors, and NMDA receptors (147, 148). The expression and function of these receptors on microglia have important implications for the onset and progression of neuroinflammation. The activation of NMDA receptors is closely associated with the release of proinflammatory factors such as NO, TNF-α, and IL-1, and excessive release of these factors exacerbates neuroinflammation and neuronal injury (149). In addition, NMDA receptors promote pro-inflammatory responses through poly ADP-ribose polymerase-1 (PARP-1) and transmembrane protein 2 (TRMP2) signaling pathways (10). It was shown that the NMDA receptor inhibitor MK801 was able to alleviate the proinflammatory polarization of microglia (11). In addition, in a mouse model of AD, the activation of AMPA receptors under non-inflammatory stimulus conditions enhances microglia phagocytosis. However, with the progression of the disease, overactivation of AMPA receptors causes overproduction of inflammatory factors and exacerbates neuroinflammation (150). These findings suggest that the role of ionotropic glutamate receptors in AD pathology is double-edged, with mild activation potentially contributing to neuroprotection and excessive activation potentially exacerbating neurological damage.

Metabotropic glutamate receptors, functioning as G protein-coupled receptors (GPCRs), initiate intracellular signaling cascades without directly forming ion channels. The expression and activation of mGluR2/5 on microglia significantly influence the inflammatory response. mGluR2 activation promotes the release of inflammatory cytokines, contributing to neurotoxicity, which is inhibited by 2S,3S,4S-2-methyl-2-(carboxycyclopropyl) glycine (MCCG) (151). In contrast, mGluR5 activation using the selective agonist (RS)-2-chloro-5-hydroxyphenylglycine (CHPG) reduces TNF-α secretion and exhibits neuroprotective effects (26). mGluR5 antagonism increases endoplasmic reticulum stress and mitochondrial dysfunction, promoting a pro-inflammatory state in microglia (152). A recent study demonstrated that genetic deletion of mGlu5 exacerbates neurodegeneration; mGlu5 knockout mice displayed increased neuronal loss and microglial activation, as well as accelerated neurodegeneration compared to wild-type mice (27). Enhanced mGlu5 signaling in neurodegenerative diseases may prevent neuronal loss, but its potential to ameliorate cognitive function warrants further investigation (153). Collectively, glutamate receptors on microglia play a dual role in AD pathogenesis. In the early stages of the disease, moderate glutamate receptor activation may contribute to microglia phagocytosis and promote Aβ clearance, thereby exerting neuroprotective effects. However, as the pathology progresses, excessive activation of glutamate receptors may lead to the release of excessive inflammatory factors from microglia, exacerbating neuroinflammation.

### 3.13 Purinergic receptors

These include P1 and P2 receptors, which are crucial purinergic signaling mediators and have a significant impact on AD pathogenesis (154). Microglia express ionotropic P2X receptors, especially P2X4R and P2X7R, as well as metabotropic P2Y receptors, which constitute the focus of studies on AD pathology (155, 156). Interestingly, activation of the P2X4 receptor enhances histone B (CatB) activity in lysosomes and promotes degradation of apolipoprotein E (ApoE), which is closely linked to Aβ clearance. In the APP/PS1 mouse model, deletion of the P2X4 receptor led to an increase in intracellular and secreted ApoE, as well as a decrease in the level of the soluble microaggregate Aβ1-42 peptide and amelioration of spatial memory deficits. These findings suggest microglial P2X4 promotes lysosomal ApoE degradation and indirectly alters Aβ peptide clearance (28). Although P2X7R is neuroprotective in physiological states, P2X7R overexpression stimulates neurodegenerative changes and promotes neurotoxic effects. The importance of P2X7R in AD pathology is emphasized by its reported upregulation in the vicinity of Aβ plaques in animal models of AD and in AD patients (157). The P2X7R activation induces potassium efflux and, in turn, activates NLRP3 inflammasome vesicles and promotes IL-1β release (158). Released interleukins, in turn, induce pyroptosis. A recent study unexpectedly found that Aβ regulates microglia migration by affecting elevated extracellular ATP concentration and activating P2X7R, leading to the accumulation of microglia in the vicinity of senile plaques, and also regulates the ability of microglia to phagocytose Aβ (159). Targeted silencing of P2X7R expression by RNA interference technology reduces P2X7R levels in the AD nervous system, decreases β-amyloid deposition and neuronal apoptosis, and ameliorates neurodegenerative pathological changes and learning and memory abilities in AD mice. This suggests that P2X7R is expected to be an effective therapeutic target for RNA interference in the treatment of AD (160). In addition, the interaction of P2X7R with tau protein pathology has been investigated. Inhibition of P2X7R may reduce the disease phenotype in a mouse model of tauopathy (P301S mice) by inhibiting the release of microglia exosomes (161). Further mechanistic studies showed that deletion of the P2X7 receptor, while having a positive effect on tau protein phosphorylation, ameliorates the neuroinflammatory response primarily by reducing microglia activation and decreasing the production of associated inflammatory markers (29). This suggests that P2X7 receptors are not only involved in the pathologic process of tau proteins but also play an important role in the pathology of AD by regulating the activation state of microglia and inflammatory responses. Taken together, the regulation of P2X7 receptors may provide new therapeutic strategies for restoring the normal function of microglia, attenuating neuroinflammation, promoting Aβ clearance, and inhibiting tau protein pathology.

### 3.14 CX3C chemokine receptor 1

CX3CR1 is a chemokine receptor specifically expressed by microglia in both human and mouse brains (162). Its binding to its ligand, CX3CL1 (also known as fractalkine), is critical for microglial homeostasis and function (163). The CX3CR1/CX3CL1 system has a dual effect on the pathologic manifestations of AD (164). For example, CX3CR1 knockout models show reduced amyloid pathology but exacerbated tau pathology, highlighting the dual role of CX3CR1 in AD. Elimination of CX3CR1 in amyloid-depositing mouse models resulted in reduced Aβ deposition, likely due to increased phagocytosis by activated microglia (165). Interestingly, in APP/PS1 mice, loss of CX3CR1 in a gene dose-dependent manner reduces Aβ aggregation (30).

Conversely, studies focusing on tau pathology have shown that CX3CR1 deletion exacerbates tau hyperphosphorylation and pathology. For example, in hTau mice, CX3CR1 deletion leads to increased tau hyperphosphorylation, greater microglial activation, and inflammation, resulting in further memory impairment (31). Notably, Bolos et al. highlighted an interesting mechanism of competition between tau proteins and CX3CL1, the natural ligand of CX3CR1. The authors showed that the amount of tau internalized is reduced in the presence of CX3CL1, emphasizing that microglia strongly influence tau internalization through CX3CR1 (166). These findings suggest that while CX3CR1 inhibition enhances microglial phagocytosis of Aβ, it may also promote tau hyperphosphorylation and aggregation by reducing tau internalization. Future studies should conduct an in-depth examination of the coexistence of Aβ and tau pathologies in Alzheimer’s disease models to elucidate the role of CX3CR1 in AD pathogenesis.

### 3.15 Colony stimulating factor 1 receptor

CSF1R is a receptor tyrosine kinase belonging to the platelet-derived growth factor receptor (PDGFR) family (167). In the CNS, CSF1R is predominantly expressed on microglia. In the adult brain, microglia are entirely dependent on CSF1R signaling for survival, making CSF1R inhibitors effective tools for microglial depletion (168).

It has been reported that microglial clearance of Aβ declines with age and the progression of AD pathology, leading to plaque formation and subsequent, unresolved inflammatory reactions (95). In the late stages of AD, microglial depletion may be an effective therapeutic option, with CSF1R inhibitors serving as a means to deplete microglia. However, microglial depletion with a CSF1R inhibitor after plaque formation does not alter β-amyloid levels or plaque load, but it prevents synaptic and neuronal loss at a late stage of pathology (169). In a later study, The effect of microglia on plaque formation was investigated by Spangenberg et al., who studied the impact of microglial depletion on neuron-derived Aβ aggregation using the CSF1R inhibitor PLX5622 in a mouse model of AD (5xFAD). They found that microglial depletion prevented the formation of plaques in the parenchymal space (32). The authors suggest that neuron-derived Aβ is internalized and aggregated within microglia, contributing to the initial formation of plaques. Additionally, microglia promote tau propagation through exosomal secretion during the early stages of AD. Depleting microglia with a CSF1R inhibitor inhibits tau propagation in mouse models (33). These results suggest that inhibiting CSF1R signaling alters the role of microglia in plaque formation and reduces tau propagation.

### 3.16 Adiponectin receptors

AdipoR1 and AdipoR2 are the two main adiponectin receptors; their expressions in the brain are mainly concentrated on cell types such as microglia, astrocytes, and neurons (170, 171). AdipoR1 expression in microglia is associated with inflammatory regulation. *In vitro* experiments have shown that the knockdown of AdipoR1 in BV2 microglia enhances the ability to release pro-inflammatory cytokines induced by Aβ (34). *In vivo* studies confirm this, showing that AdipoR1 knockdown mice experience memory deficits and exhibit Alzheimer’s-like symptoms, such as neuronal oxidative stress, insulin resistance, and heightened neuroinflammation (172). Adiponectin, which acts via AdipoR1, lessens the inflammatory response to Aβ in microglia, but this protective effect is negated when AdipoR1 is downregulated (34). AdipoRon, a small molecule adiponectin receptor agonist, reduces inflammation by decreasing microglial activation and lowering cytokine levels (173). These findings suggest that AdipoR1 in microglia may be involved in the pathologic process of AD and that its changes may have important implications for altered cognitive function in AD. Since AdipoR1 and AdipoR2 are co-expressed in cells, it can be hypothesized that AdipoR2 may also be involved in the pathological process of AD through similar or different mechanisms (174). In conclusion, AdipoR1 may be involved in the pathogenesis of AD through the regulation of the microglial inflammatory response, but the role of AdipoR2 remains to be investigated.

## 4 Receptors related to synaptic plasticity of neuron

**Figure 2:**
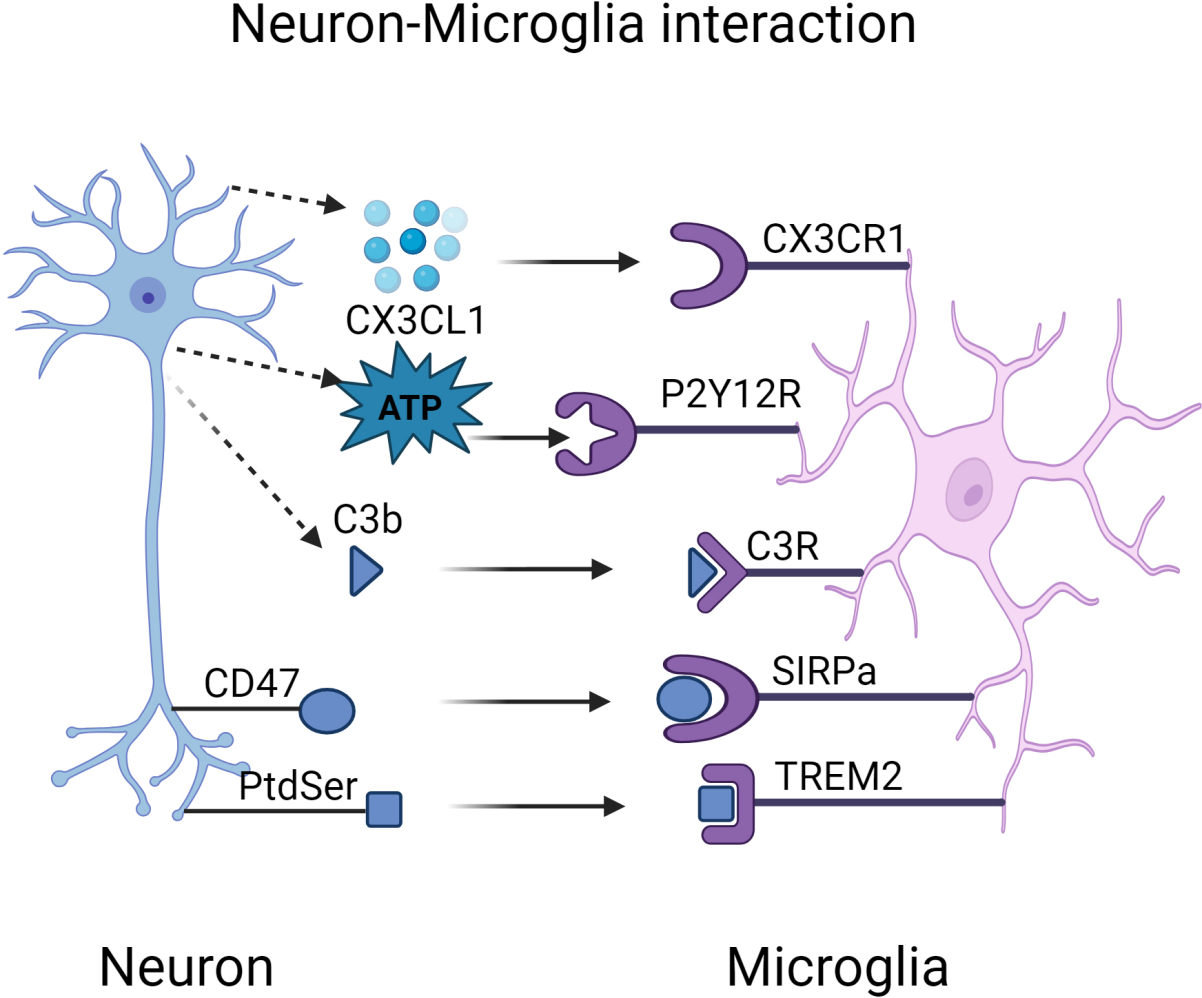
Interactions Between Microglial Receptors and Neurons in Alzheimer’s Disease Pathogenesis. Created in BioRender.com. Interactions between microglial receptors and neurons collectively regulate the synaptic strength of neurons. During the pathogenesis of Alzheimer’s disease, microglia fail to effectively clear excessive Aβ and tau aggregates, which chronically activate and damage both neurons and microglia. Abnormal release of ‘find-me’ signals, such as CX3CL1 and ATP, leads to a delay in the synaptic pruning process. Aβ induces the overexpression of ‘eat-me’ signals, including PtdSer-TREM2 and iC3b-CR3, resulting in excessive synapse elimination. CD47, a normally expressed ‘don’t-eat-me’ signal, exhibits reduced expression in AD, leading to an increased phagocytic capacity of microglia, which may exacerbate synaptic loss. ATP, adenosine triphosphate; CX3CL1, C-X3-C motif chemokine ligand 1; CD47, cluster of differentiation 47; CR3, complement receptor 3; PtdSer, phosphatidylserine; SIRPα, signal regulatory protein α; TREM2, triggering receptor expressed on myeloid cells-2.

Synapse elimination is a finely regulated physiological phenomenon during the development and maturation of the CNS that involves the removal of nonfunctional or redundant synaptic connections. This process is critical for optimizing the efficiency of neural networks (175). Microglia play an important role in this process, both by removing apoptosis and by directly influencing neuronal regeneration. For example, among all neurons produced during neural development and adult neurogenesis, some cells then undergo programmed cell death and are rapidly cleared by microglia (176). Synaptic damage is an early pathological feature of AD, and its development is closely related to cognitive dysfunction (177). Microglia are key to synapse clearance and formation, and their phagocytosis is regulated by the activity-dependent regulation of the immune signaling proteins ‘eat me’ and ‘don’t eat me’, which determine which synapses will be cleared, especially those inactive synapses (178). Although physiological phagocytosis of synapses is essential for the fine-tuning of experience-dependent neural networks, excessive synaptic clearance may lead to the loss of live neurons and synapses, which can trigger neurodegeneration. This pathological phagocytosis can be triggered by a number of factors, including aberrant release of ‘find-me’ signaling, overexpression of ‘eat-me’ signaling, or absence of ‘don’t-eat-me’ signaling (179) (**Figure 2**).

### 4.1 ‘Find-me’ signaling

#### 4.1.1 CX3CL1-CX3CR1

CX3CL1, a neuron-derived chemokine, activates microglia and guides their migration to specific synaptic regions by binding to its receptor, CX3CR1. This interaction is crucial for microglia to recognize and respond to synaptic pruning signals. Acting as a soluble “find-me” signal, CX3CL1 induces microglial-mediated synaptic pruning, and CX3CR1 knockdown makes microglia unresponsive to CX3CL1’s chemotactic effects (180). In developing hippocampal and barrel cortex regions, CX3CR1-deficient mice show a transient delay in microglia recruitment to synapse-rich areas and a delay in synaptic functional maturation (181). Over the long term, CX3CR1-deficient mice exhibit defects in social interaction and functional synaptic connectivity (182). A similar phenomenon occurs in the absence of CX3CL1, where thalamocortical synapse elimination is impaired due to defective microglial phagocytosis in CX3CL1-deficient mice (183). These findings underscore the critical role of the CX3CL1/CX3CR1 axis in regulating synaptic plasticity.

#### 4.1.2 ATP-P2Y12 receptor

The P2Y12 receptor is a G protein-coupled receptor that is expressed on microglia and is involved in synaptic surveillance, pruning, and clearance (184). In the early stages of neurodevelopment, microglia monitor immature neurons and regulate neurodevelopmental processes through P2Y12R-mediated somatic interactions (185). The absence of P2Y12R disrupts these functions, resulting in the aberrant development of cortical cellular structures (186). This somatic connection is a key mechanism for microglial monitoring and control of neural development. Similarly, knocking down P2Y12R in adult mice after normal development reduces microglial involvement in synaptic pruning, thereby impacting neural network refinement and plasticity (187).

ATP-induced P2Y12R chemotaxis is considered an early response to minor pathologic signs (188, 189). For instance, it directs microglia to overactive neuronal axons, protecting them through cell-cell interactions (190). In AD, ATP released by damaged neurons activates P2Y12 receptors on microglia, triggering their migration to the site of damage to engage in the clearance of damaged synapses and neurons (191).

### 4.2 ‘eat-me’ signaling

#### 4.2.1 Complement pathway

In the adult CNS, expression of the complement component C1q is normally maintained at low levels to ensure the stability of neural circuits. However, in brain injury or pathological states such as Alzheimer’s disease, the C1q expression is significantly upregulated in the injured region, and By integrating multi-omics analyses, it was shown that complement proteins (e.g., C1q) are co-localized with the postsynaptic PSD95 dot in an AD mouse model, which suggests that C1q can act as a molecular label on synapses in preparation for microglia phagocytosis (192). Microglia express high levels of the C1q receptor (C1qR) and complement component 3 receptor (CR3). C1q plays a unique role in the nervous system as an initiating protein of the classical complement cascade reaction. At neuronal synapses, the activation of C1q can trigger activation of CR3 receptors, which in turn facilitates synapse recognition and clearance by microglia (193). Notably, microglia are the only cell type in the brain that expresses CR3 (194), a property that confers them a unique function in synapse clearance. In mouse models of AD, activation of the complement system was found to precede the formation of amyloid plaques (195), and the finding that microglia are involved in synaptic loss through a complement-mediated mechanism underscores the importance of the complement system in the early pathological process of AD. By inhibiting key components of the complement cascade reaction, such as C1q, C3, or CR3, on microglia, they can significantly rescue synapse density and prevent microglia-mediated synapse loss (196). In addition, the use of C1q-blocking antibodies is a potential intervention strategy that can reduce microglia overactivation and synaptic damage (197). These findings suggest that C1q-blocking antibodies may play an important role in the treatment of AD by reducing microglia overactivation and synaptic damage, thus providing a potential therapeutic strategy for AD treatment.

#### 4.2.2 PtdSer-TREM2

Phosphatidylserine (PtdSer) is a membrane phospholipid, and during apoptosis, externalization of PtdSer is a clear ‘death signal’ that is irreversibly exposed at the cell surface and directs synaptic pruning (198). In the Aβ model of AD, the externalization of PtdSer is associated with early dysregulation of synaptic function (199). Microglia exhibit selective phagocytosis of damaged synapses expressing PtdSer. Aβ oligomers induce synaptic hyperactivity and contribute to the externalization of PtdSer, a classic ‘eat me’ signal (200). These apoptosis-like spines are recognized and phagocytosed via TREM2 receptors on microglia, thereby ameliorating Aβ oligomer-induced synaptic hyperactivity. Higher levels of apoptotic-like synapses have been observed in mouse models and in humans carrying loss-of-function variants of TREM2 (67). The deletion of TREM2 results in impaired synaptic elimination accompanied by increased excitatory neurotransmission (201). Furthermore, the synaptic removal by microglia in mouse models of AD tauopathy has been investigated. A study using co-cultures of BV2 microglia and neurons expressing phosphorylated tau demonstrated that microglia can engulf intact, living neurons by recognizing exposed PtdSer (202). Recent studies have further revealed the potentially beneficial role of TREM2 in the early stages of AD. A study by Rueda et al. found higher levels of uncleared apoptosis-like synapses in mouse and human brains from patients with loss-of-function mutations in TREM2. This suggests that the removal of overactive synapses by microglia in AD is important for maintaining normal nervous system function (203). Future studies need to explore in depth the interaction between PtdSer and TREM2 in AD and its potential application in disease treatment.

### 4.3 ‘Don’t eat me’ signaling

#### 4.3.1 CD47-SIRPα

CD47 is a widely expressed ‘don’t eat me’ signaling protein that exerts its protective effects by binding to the signal-regulated protein alpha (SIRPα) receptor on microglia. SIRPα is expressed at high levels on microglia during critical periods of brain development and helps to inhibit synaptic hyperphagy (204, 205), The presence of CD47 may protect highly active synaptic populations from becoming targets of microglia-mediated pruning processes (206). In the context of AD, decreased expression of CD47 leads to increased phagocytosis of synapse by microglia, which may exacerbate synaptic loss. A study by DeVries et al. found an association between elevated C1q at PSD95-positive synapses and decreased CD47 in age-related cognitive impairment. In addition, they observed reduced CD47 RNA expression in neurons, suggesting that aging neurons may be defective in producing the protective signal CD47 (207). These findings emphasize the importance of CD47-SIRPα signaling as a ‘don’t eat me’ signal during brain development and neurodegeneration. This signaling mechanism is essential to protect synapses from microglia-mediated excessive synaptic pruning.

## 5 Therapeutic strategies based on microglia receptors

Currently, there are a large number of preclinical studies demonstrating the potential efficacy of some microglia receptor families in the treatment of AD, but these studies have taken less account of the complexities involved in targeting microglia receptors for therapy. These complexities might come from the spatial and developmental heterogeneity of microglia revealed by recent studies using single-cell RNA sequencing (208). This heterogeneity increases the complexity of therapeutic strategies for microglia receptors. The discovery of disease-associated microglia suggests that microglia may play diverse functions at various stages of AD, and their activation state and function may be influenced by multiple factors (122). Therefore, a deeper understanding of the molecular mechanisms underlying microglia heterogeneity is important for the development of new therapeutic approaches. Considering the complexity of microglia receptors in the determination of treatment strategy for AD would be of great importance. Nonetheless, we reviewed current studies of microglia receptor-based therapeutic strategies, including preclinical studies, and mostly studied receptors.

### 5.1 CSF1R inhibitor

A hallmark of AD is the hyperproliferation and activation of microglia. CSF1R, essential for microglial survival and proliferation, is a target for antagonists that have shown promise in preclinical AD models for preventing cognitive decline. For example, in APP/PS1 mice, CSF1R inhibition with the oral tyrosine kinase inhibitor GW2580 reduced plaque-associated microglia and normalized behavior (209). Similarly, in 5XFAD mice, short-term (10 days) CSF1R inhibition with PLX5622 (Plexxicon Inc.) promoted homeostatic microglia retention and decreased inflammasome activation (210). In a model of tau disease, Edicotinib, a CSF1R inhibitor, effectively reduced microglial inflammation and tau-induced neurodegeneration and improved cognitive function in P301S mice (211). These observations suggest that reducing dysfunctional microglia may be an effective strategy for modulating AD pathology.

### 5.2 TREM2 activation

The activation of TREM2, a key microglia surface receptor, has been shown to promote Aβ clearance and improve cognitive function in AD mouse models (212). Activation of TREM2 through the use of agonist monoclonal antibodies enhances its downstream signaling, thereby increasing phagocytosis and anti-inflammatory effects in microglia. This strategy has received much attention in the treatment of Alzheimer’s disease.

AL-002 (NCT04592874), a monoclonal antibody against TREM2 developed by Alector for early AD treatment, has recently completed Phase 2 clinical trials, with results pending publication. In a previous Phase 1 clinical trial (NCT03635047), AL-002 demonstrated safety and good tolerability (213). It also decreased filamentous Aβ plaques and attenuated the microglial inflammatory response in preclinical models (214). In addition, Denali Therapeutics and Takeda have partnered to develop an antibody called DNL919 (ATV: TREM2), which is designed to activate the TREM2 receptor through intravenous administration to improve TREM2 function for therapeutic use in AD. A Phase 1 clinical trial of DNL919 (NCT05450549) was successfully initiated in July 2022; however, the development of the drug was terminated in 2023 due to safety concerns in healthy volunteers. Besides, a study exploring the effects of DNL919 on human-induced pluripotent stem cell (iPSC)-derived microglia found that it promoted the proliferation of these key immune cells and improved their metabolism (215). Overall, DNL919 activated TREM2 and improved microglia function.

While the TREM2 activation strategies show great potential in AD treatment, studies have also shown that their efficacy may be limited by disease stage. The benefits of enhanced TREM2 signaling may be time-dependent and more pronounced in early AD pathology (216). Therefore, future studies need to further explore the optimal timing and conditions for TREM2 activation to achieve more effective interventions in AD.

### 5.3 PPARγ agonists

PPARγ agonists have demonstrated some potential in the treatment of AD and have been shown to be associated with suppression of pro-inflammatory cytokines and amelioration of inflammatory diseases (217). One study further elucidated that PPARγ agonists improve cognitive function by inhibiting inflammation and reducing apoptosis, an effect mediated by activation of the PPARγ/NF-κB signaling pathway, suggesting that PPARγ agonists may serve as potential therapeutic agents for the treatment of AD (218). Notably, T3D Therapeutics, Inc. has developed a drug called T3D-959, a dual PPAR δ/γ agonist that showed signs of improvement in cognitive function tests in patients with mild to moderate AD in an exploratory phase IIa study (NCT04251182) (219). Although PPARγ agonists show potential in AD treatment, most of the current studies are still in the preclinical phase, and more clinical studies are needed to validate their safety and efficacy.

### 5.4 P2X7R antagonists

The presence of the P2X7R isoform on the surface of microglia, which has dual functions of neuroprotection and neurodegeneration, has emerged as a potential therapeutic target for AD (220). As mentioned previously, inhibition or knockdown of P2X7R has been shown to attenuate P2X7R-mediated inflammatory responses and ameliorate neuropathological changes associated with AD in a variety of animal models of AD. Notably, P2X7R activation usually occurs when extracellular ATP levels reach pathological concentrations, suggesting that P2X7R inhibition may be particularly effective in pathological states (221). Brilliant blue G (BBG), a known P2X7R antagonist, has been used in the treatment of neurological disorders due to its ability to penetrate the blood-brain barrier and to show effectiveness against P2X7R in different species, thus showing potential applications in the treatment of neurological disorders (222, 223). BBG attenuates Aβ-induced inflammatory response and induces microglia to exert anti-inflammatory effects (224). In addition, several other drugs and compounds are being investigated as P2X7R antagonists to explore their potential in AD therapy. For example, AZ10606120 is another P2X7R antagonist that has shown inhibitory effects on neuroinflammation in preclinical studies (225). Anti-AD drugs targeting P2X7R must balance their selectivity, bioavailability, blood-brain barrier permeability, and toxicity. Future studies need to further explore the pharmacological properties of these drugs, as well as their specific mechanisms of action in AD pathology, in order to achieve more effective and safer treatments for AD.

### 5.5 RAGE inhibitors

RAGE is a multi-ligand receptor associated with a variety of pathological processes, including inflammation, cell signaling, and cell survival. Azeliragon (TTP488) and FPS-ZM1 are two promising inhibitors of RAGE that intervene in the pathological processes of AD through different mechanisms. Azeliragon (TTP488) is an orally bioavailable small-molecule RAGE antagonist that inhibits inflammatory signaling by blocking the binding of RAGE to its ligands, including Aβ, advanced glycosylation end-products (AGEs), S100 proteins, and high mobility group protein 1 (HMGB1) activation (226). In phase 2b (NCT00428701) clinical trials, Azeliragon showed the potential to reduce brain Aβ plaque levels, increase plasma Aβ concentrations, decrease inflammatory cytokine levels, and slow cognitive decline (227). Azeliragon is currently undergoing a Phase 3 clinical trial (NCT02080364) for AD to further evaluate its effects on cognitive function in patients with mild AD. FPS-ZM1 (4-chloro-N-cyclohexyl-N-benzylbenzamide) is another small-molecule RAGE inhibitor, which works by binding to the RAGE receptor to inhibit Aβ40 and Aβ42 production and deposition, showing potential effects on AD treatment (228). Notably, the mechanism of action of Azeliragon and FPS-ZM1 is not limited to AD treatment, but Azeliragon has also shown potential to inhibit tumor progression and metastasis in other disease areas, such as the treatment of triple-negative breast cancer (229).

### 5.6 NLRP3 inhibitors

Currently, inhibitors targeting the NLRP3 inflammatory vesicle can be categorized into two main groups: small molecule compounds and natural products, which have shown potential therapeutic potential in preclinical studies. Small molecule inhibitors such as MCC950, a specific NLRP3 inflammasome inhibitor, have shown therapeutic efficacy in several preclinical models. MCC950 reduces inflammatory responses and improves cognitive function by inhibiting NLRP3 inflammasome activation (230). Although MCC950 has been tested in a number of clinical trials, specific clinical trial results have not yet been published. In addition, Qiang Li’s team at Tongji University reported UK5099 as a novel inhibitor of NLRP3 inflammatory vesicles. UK5099 was found to be a potent NLRP3 inhibitor, which effectively inhibited NLRP3 inflammasome-mediated IL-1β production in both *in vitro* and *in vivo* experiments (231). The inhibitory ability of UK5099 is comparable to that of RRx-001, which has entered phase III clinic, and has a favorable safety profile, showing its potential for clinical application. In addition, some natural products, such as resveratrol, radicicchioidin, aloe vera, and curcumin, have been found to have the ability to inhibit the activation of NLRP3 inflammatory vesicles (232). These natural products may inhibit the assembly or activation of inflammatory vesicles through different mechanisms, showing potential as therapeutic candidates for AD treatment. Currently, research and development of NLRP3 inflammasome inhibitors is actively underway, and some candidate molecules have demonstrated therapeutic potential. However, the clinical efficacy and safety of these candidate molecules still need to be verified by further clinical trials.

## 6 Conclusion and perspectives

The role of microglia receptors has received increasing attention in AD research as immunomodulatory therapy via microglia receptors has shown great potential for treatment. To further investigate the advantages of immunomodulatory therapy, it is crucial to define the roles of microglial receptors, including those involved in phagocytosis and inflammatory regulation, as well as the associated signaling pathways. Nonetheless, current research on these receptors is still in the exploratory phase. Epigenetic change in microglia is greatly influenced chronologically and spatially (233). The functional roles of microglia receptors may vary with brain regions and physiological status (234).

The double-edged function of certain receptors (e.g., CD36) and the impact on the immune system should be carefully considered when developing therapies. For example, TREM2 receptors play a role in regulating microglia phagocytosis and attenuating AD-associated pathological changes, but their role in the tumor microenvironment should not be overlooked (235, 236), especially when treatment with TREM2 agonists could stimulate tumor growth. Future studies need to further explore the spatiotemporal dynamics of AD microglia receptors and how these properties can be utilized to provide new strategies for AD therapy.
